# Failure Progress of 3D Reinforced GFRP Laminate during Static Bending, Evaluated by Means of Acoustic Emission and Vibrations Analysis

**DOI:** 10.3390/ma8125490

**Published:** 2015-12-14

**Authors:** Mateusz Koziol, Tomasz Figlus

**Affiliations:** 1 Faculty of Materials Engineering and Metallurgy, Silesian University of Technology, ul. Krasinskiego 8, 40-019 Katowice, Poland; 2 Faculty of Transport, Silesian University of Technology, ul. Krasinskiego 8, 40-019 Katowice, Poland; tomasz.figlus@polsl.pl

**Keywords:** glass fibre reinforced polymer laminate, 3D reinforcement, failure analysis, signal analysis, vibrations analysis, acoustic emission

## Abstract

The work aimed to assess the failure progress in a glass fiber-reinforced polymer laminate with a 3D-woven and (as a comparison) plain-woven reinforcement, during static bending, using acoustic emission signals. The innovative method of the separation of the signal coming from the fiber fracture and the one coming from the matrix fracture with the use of the acoustic event’s energy as a criterion was applied. The failure progress during static bending was alternatively analyzed by evaluation of the vibration signal. It gave a possibility to validate the results of the acoustic emission. Acoustic emission, as well as vibration signal analysis proved to be good and effective tools for the registration of failure effects in composite laminates. Vibration analysis is more complicated methodologically, yet it is more precise. The failure progress of the 3D laminate is “safer” and more beneficial than that of the plain-woven laminate. It exhibits less rapid load capacity drops and a higher fiber effort contribution at the moment of the main laminate failure.

## 1. Introduction 

Knowledge of the failure progress is especially important for the responsible elements exposed to unexpected loads (e.g., bridge bearing elements [1]), and it should be a complement to the basic mechanical characteristics of such elements. Glass fibre reinforced polymer (GFRP) composite is a specific material with respect to its failure progress [2,3]. It contains a brittle matrix with certain (mainly elastic) deformation abilities and brittle fibers as well. That is why model descriptions applied for laminates are usually the ones for brittle materials [4], especially multi-phase ones [5,6]. However, GFRP laminates also show pseudo-plastic properties [2,7]. Therefore, in some analysis cases, the approach used is the one for metals [8,9] or thermoplasts [10,11]. Laminates exhibit a very stochastic behavior in the experimental analysis. This stochasticity of the laminates’ properties is influenced by the technological factors connected with the imperfection of the reinforcing fabrics [12,13] or the errors occurring in the manufacturing process [14,15]. 

Failure initiation is probably the most important issue concerning failure analysis. After the failure appears, the material is permanently weakened. Therefore, in composite constructions, it is crucial to detect the beginning of the failure process [1,16]. This can be achieved, among others, by means of diagnostic tests of effort in composite constructions. These include ultrasound methods [17,18] and thermography [19]. An even better and more up-to-date method is continuous monitoring of the state of the laminate’s structure with the use of interlaminarly-placed detector systems. An example can be acoustic emission (AE) sensors [20], optical fiber sensors [21,22] and piezoelectric sensors [23,24]. There is a tendency to expand this methodology by the application of methods used in other areas, such as resistance analysis [25], thermal analysis [26], surface state analysis [27,28] and vibration signal analysis [29]. 

Identification of individual failure mechanisms is especially important in 3D laminates. This is because this type of laminate is usually applied in very responsible constructions, requiring improved delamination resistance [23,30]. Failure progress analysis may show the efficiency of this improvement, especially in bending [31] and interlaminar tension (Mode I) [32]. However, even more studies were performed on tension, among others, [31,33,34].

A separate issue is the post-process analysis of laminate failure, based on the classic methods used in materials science [35,36]. It is supposed to help with the modelling of the laminate failure prediction, which is still under the consideration of science [37,38]. For laminates, the modelling methods used mainly in the case of brittle materials are adapted [39], but also those applied for nanomaterials [40,41], as well as behavior modelling by long-term [42] or cyclic [43,44] loads. In order to predict and model the above, it is necessary to understand the structure of the material in laminates; methods similar to those used for the classic materials are used [45]. Good knowledge of the failure mechanics makes it possible to find solutions that prevent damage and catastrophic failure, e.g., self-healing nanocomposites [46], composites with a modified structure [47,48] or precise tailored structures [49,50]. 

Identification of the effects of failure after some damage is especially important for translaminarly-reinforced laminates. This gives information about the efficiency of the improvement in delamination resistance given by the translaminar reinforcing elements. In this case, the application of nanotubes for damage characterization of 3D braided composites (*in situ* sensing) seems very interesting [51]; however, this demands additional research to be well validated. The most popular method probably remains the AE method combined with imaging analysis [52], which, among others, has been applied by the authors in the earlier research works [53,54,55] and in the current study, as well. 

This work is a continuation of the authors’ earlier studies concerning vibration analysis [53,54] and AE [55] during laminate failure in bending. The authors established there that the main cause of laminate failure during bending is delamination, which was clearly proven in acoustic emission tests [55], as well as vibration analysis [53,54]. As a continuation of the studies, the authors propose a new idea of analyzing the 3D woven laminate failure progress. It consists of applying the energy of acoustic events originating from the fibers’ fracture and those originating from the matrix fracture as the separation criterion. This is an innovative and simplified method of separating the acoustic emission signal, already used in [55] for stitched laminate. The problem with the method is the fact that energy is an extensive parameter (dependent on the dimensions of a specimen). The natural factor for analyzing the breakage of individual components should be frequency. However, practice showed that it is very difficult to perform an efficient separation analysis with the use of frequency [56]. In some cases (bending, glass fiber reinforcement), it became practically impossible [55]. 

In order to verify this new approach, an additional energy analysis of the vibration signal was performed. The method had its value already confirmed in the assessment of laminate failure progress, among others, in [53,54]. To analyze the vibration signal efficiently, the proper signal measure must be used [57,58]. Kurtosis was used as the measure of the signal’s character changes in this study. 

## 2. Experimental Section 

For the purpose of the study, laminates on the basis of two types of preforms were prepared:

- Classic preform (only for AE tests, comparison purposes): 10 layers of plain-woven glass fabric (E glass), produced by KROSGLASS (Krosno, Poland), areal mass 350 g/m^2^ (total of 3500 g/m^2^), preform thickness 2.65 ± 0.02 mm; preform for classic laminate.
- 3D preform (non-crimp, E glass), produced by 3TEX (Rutherfordton, NC, USA), woven of 7 layers: 4 in the weft direction and 3 in the warp direction, areal mass 3280 g/m^2^, fabric thickness 2.65 ± 0.02 mm. The preliminary analysis showed that the structural model of this fabric is the most appropriate from among the currently available free trade fabrics for the comparison with the classic preform; preform for 3D laminate.

The matrix of both laminates was polyester resin ESTROMAL 14 LM, produced by LERG S.A. (Pustkow, Poland) + hardening agent METOX 50 (a solution of methyl ethyl ketone in dimethyl phthalate). 

The laminates were formed as flat panels (250 mm × 350 mm), by the vacuum assisted resin infusion (VARI) method. After initial curing, the panels were left at room temperature for a period of about 20 h, for the main hardening. Next, they were additionally cured at 55 °C for 4 h and subsequently left for at least 70 h at room temperature (acclimatization). The samples were cut out from the plates with the use of a rotating blade. 

Concerning the anisotropy of the 3D fabric, special designations are used in the study. For the loading in the direction along the translaminar interweave strands in the 3D laminate (warp direction), an additional designation “*W*” will be used, whereas for the direction transverse to the interweave strands (weft direction), the designation “*P*” will be used. 

Within the frames of the study, two separate types of registration were applied: Static bending tests with AE registration and static bending tests with vibration registration. 

For both types of registration, three-point bending was performed in accordance with the standard PN-EN-ISO 14125 [59]. A constant supporting bars spacing/sample thickness ratio was set at 30/1; supporting bar spacing was set at 80 mm. The recording of the value of deflection and force was performed with the sampling frequency *f*_max_ *=* 10 Hz. The bending tests were conducted on a set of 4 samples for each laminate type (classic, 3D-*W*, 3D-*P*). The preliminary analysis made it possible to state that the courses of the characteristics of particular samples in both groups are similar. In order to meet the limited size of the publication and to present a clear argument, the analysis of the vibration signal involved only 1 representative sample from each set. Those samples were selected from the set whose maximal stress was the closest to the mean value for the given population. 

**Figure 1:**
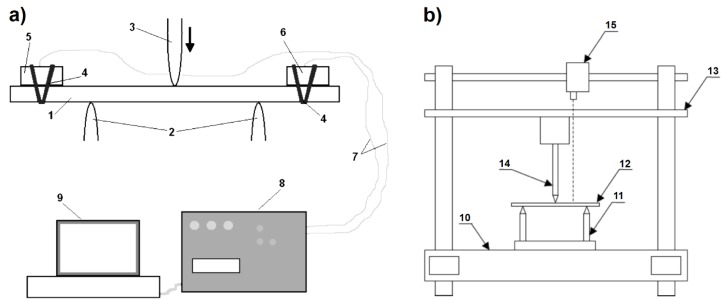
Scheme of the measuring devices used for signal registration during 3-point bending tests: (**a**) Acoustic emission signal registration; 1, sample; 2, supports; 3, loading bar; 4, elastic fixing welts; 5, Acoustic Sensor I; 6, Acoustic Sensor II; 7, wires; 8, Vallen registrator; 9, computer; (**b**) Vibrations signal registration; 10, machine frame; 11, supports; 12, sample; 13, movable traverse with a force-measuring head; 14, loading bar; 15, laser vibrometer.

The static bending tests with AE registration were conducted on the ZWICK/ROELL 4465 testing machine (Zwick GmbH, Ulm, Germany). The spacing of the supports was 80 mm, and the deformation rate was 2 mm/min. The samples were beam shaped with a size of 160 × 35 mm. A schematic diagram of the measuring system is presented in Figure 1a. For AE signal registration, the Vallen AMS 3 device (Vallen Systeme GmbH, Icking, Germany) equipped with the Vallen VS45-H sensor (registered frequency range 20–450 kHz) and the Vallen VS375-RIC sensor (registered frequency range 250–700 kHz) was used. A computer was used to control the system and perform the final data registration. The AE sensors were fastened to the samples by means of rubber turnbuckles. The contact surfaces of the sensors with the sample were precisely covered with special vaseline, which improved the acoustic wave conduction. For the purposes of signal separation analysis, beside the laminate samples, also 9 samples of cast and cured resin (beams 230 × 30 × 5 mm; support spacing during bending at 150 mm), the pure laminates’ matrix, were tested. This was necessary to obtain data for the AE signal filtration. Supplementarily, a photographic recording of the changes taking place in the central area of the samples was made. The Sony camera with replaceable optics was used, whereas the recording was conducted at a resolution of 1920 × 1080 pixels and with rate of 25 frames/s. This enabled a thorough observation of the occurrence of the subsequent effects of failure and their time localization with an accuracy of 0.04 s. The start-up of the photo recording was synchronized with the initiation of the bending tests and the AE measurements. The quality of the obtained images was satisfyingly high. 

The static bending tests with the registration of the vibrational signal were performed with the use of a testing machine INSTRON 4469 (Instron, Norwood, MA, USA), which was additionally equipped with a vibration measuring system. The scheme of the whole measuring device is presented in Figure 1b. The method is also described in the patent application [60]. 

The samples had the shape of beams of 100 × 35 mm. A constant loading bar rate was applied for all specimens, equaling 60 mm/min. The relatively high speed (short test time) made it possible to acquire high resolution data from the vibration measurements. It also made it possible to use the units of deflection and time interchangeably (1 mm corresponds to 1 s). The measuring system was equipped with a laser vibrometer OMETRON (Ometron Ltd., Hertfordshire, UK), used to perform the non-impact measurement of the vibration velocity of different samples [61,62], also in former studies by the authors [53,54]. The laser beam was situated in a way so as to enable the measurement of the vibrations in the mid-distance between the supporting bar and the loading bar. The vibration velocity of the samples was recorded with a sampling frequency of 20 kHz. In the recording, the data acquisition card NI and the LabView software (National Instruments, Austin, TX, USA) were used, whereas the signal processing was performed with the use of MATLAB-Simulink. The tests and the signal recording were conducted till the moment of the terminal breakage of the sample. Part of the technological and methodological procedures described above (covering vibration signal registration) were formerly presented in [53]. 

## 3. Results and Discussion 

### 3.1. Acoustic Emission Results 

The main task of the first stage of the material’s failure progress analysis is to separate the signal components coming from the particular failure mechanisms. In the case of a composite laminate, there are three basic mechanisms to differentiate: fiber fracture, matrix fracture and fiber-matrix cohesion break. The latter is especially difficult to separate from the second one in spectroscopic methods, and so, they can be analyzed together. In the further part of the work, the “communized” part of the signal for those two failure mechanisms will be referred to as “matrix fracture”. 

**Figure 2:**
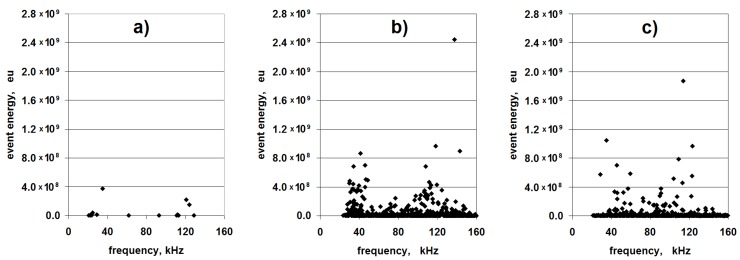
Exemplary diagrams: event energy-frequency (E-f) for: (**a**) Cured resin matrix; (**b**) Classic laminate; (**c**) 3D-*W* laminate.

The first stage of the analysis of the registered acoustic emission signal (necessary for signal separation) included creation of the event energy-frequency (E-f) diagrams for each sample. Exemplary diagrams are presented in Figure 2. 

After creation of the E-f diagrams for all of the samples, the former underwent a separative analysis. It turned out that separating the signal with the use of the frequency, most commonly used in wave spectroscopy for quality signal separation, was difficult (in fact, impossible). One should observe the diversification of the fracture signal frequency due to about a 30-times difference in the modulus of elasticity between the fiber and the resin. However, the frequency ranges of the event’s occurrence in the samples of cured resin and the composites, at about 38 and 120 kHz, were similar (Figure 2). However, an interesting regularity was established within the E-f results. In the case of all nine samples of cured resin, the maximal registered event energy equaled less than 4.0 × 10^8^ eu (eu is the referential unit of the signal energy equaling about 10^−18^ J). At the same time, in the diagrams referring to composites, the energies of some of the events significantly exceeded this value (see Figure 2a–c). 

**Figure 3:**
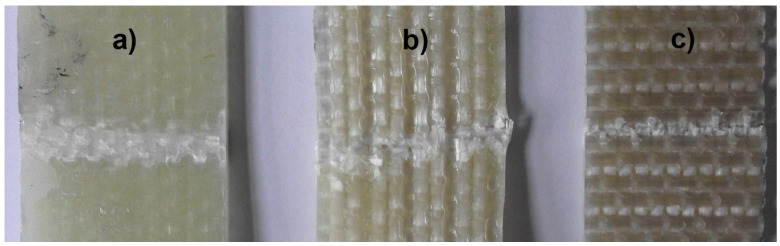
Failure after the bending tests visible on the upper surfaces of the exemplary samples: (**a**) Classic laminate; (**b**) 3D laminate *W* direction; (**c**) 3D laminate *P* direction.

In view of the above, the event energy may be used as the criterion for signal separation. Despite the fact that energy is an extensive quantity (in the analyzed case, dependent on the size of the fractured area), in the studied issue, it can be established with high certainty that the events with energy higher than 4 eu originate in the fiber fracture. The measurement for the diagram in Figure 2a was performed on a sample with a cross-section of 30 × 5 = 150 mm^2^. It can be assumed that the obtained maximal energy value (4 eu) is related to such a value of the fracturing laminate matrix material. It is improbable to obtain a similar value of the fracturing material of the laminate matrix; the total surface area of the beam cross-section equals approximately 35 × 2.65 = 92.75 mm^2^. Only during the delamination process is it possible to observe the breaking of the matrix cohesion in relatively large areas. However, these areas are also merely comparable with the surface area of the resin beam section. It should be assumed that the occurrence of a single acoustic event originating from delamination is connected with a fracture in one interlaminar area; the first failure effect occurs in the first area where the local stress exceeds the continuum strength. It is practically impossible for this to happen in a few places simultaneously. The further stages of failure progress are followed by independent acoustic signals. For the estimation of the extent of delamination, the surfaces of the laminate samples visible from the outside were measured. This was performed on the samples that had been damaged in the bending tests; exemplary images are shown in Figure 3. 

After the evaluation of the size of the failure area visible on the upper surface for each sample, it was established that the maximal surface of this area is 162 mm^2^ (for one of the classic laminate samples). This is the result after the bending tests, that is in the whole deformation range. The fracture area at the moment of its initiation was undoubtedly smaller. This result is comparable with the cured resin beam section (150 mm^2^). It should be mentioned that the delamination fracturing will be connected with a lower energy than the fracturing of the hardened resin beam section, as in some part, it consists of breaking the cohesion between the fibers and the matrix. It is only partially connected with the break of the matrix continuum. Therefore, it can be stated that the value of 4 eu constitutes the boundary value of the matrix fracture in the bent laminate beam, the limit beyond which the acoustic events are attributed to fiber fracture. This statement is the finding of the application of the event energy as the criterion of signal separation in AE. 

A serious difficulty of the “energy” approach is the impossibility to establish the fracture of elementary fibers, as well as small amounts of them. Signals coming from such events will be characterized by a relatively low energy, equaling less than the 4 eu limit. Therefore, for an accurate emission signal analysis at the initial bending stage (failure initiation), another separation method should be used; probably, involving the frequency would be necessary in some way [56]. 

**Figure 4:**
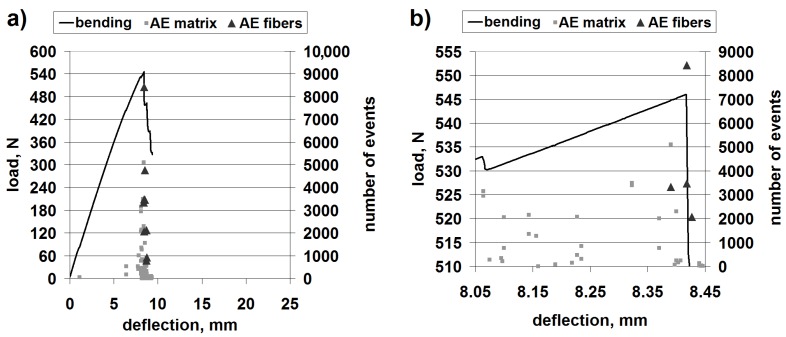
Mechanical curve and separated acoustic emission (AE) signal for the classic laminate: (**a**) The whole progress; (**b**) Magnification of the crucial fragment.

Figure 4 shows the results of a representative bending test with superimposed AE scatter diagrams, with separated points corresponding to matrix fracture and fiber fracture, for the classic laminate. 

The obtained load capacity of the beam suggests that the bending strength is of the order of 260 MPa, which constitutes a high value and proves the classic laminate to be a valuable construction material. 

**Figure 5:**
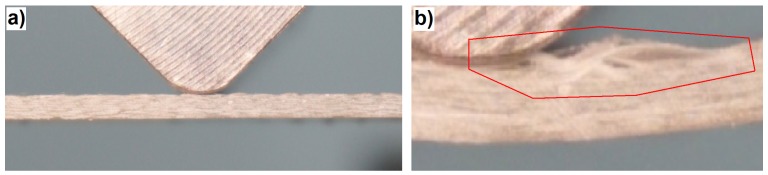
Photograph showing the main failure in the classic laminate: (**a**) Beginning of the bending tests and (**b**) 35 s of the test, right after the occurrence of the main failure.

The AE results clearly suggest that the first important failure mechanism is matrix fracture. This is in accordance with the macroscopic observations, where extensive delamination in the upper part of the beam can be seen (Figure 5). 

**Figure 6:**
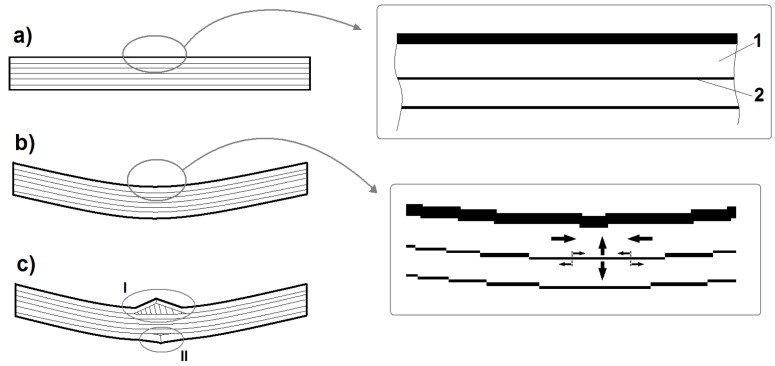
Laminate schematic diagram: (**a**) Elements of laminar structure; 1, rigid layer of reinforcement impregnated with matrix; 2, thin matrix layer between the reinforcement layers; (**b**) Schematic imaging of the forces acting on the matrix layer; stretching originating from the upper layer’s buckling trend caused by its compression; interlaminar shearing; (**c**) Schematic imaging of the laminate failure as a result of bending; delamination and buckling of one or more layers in the upper part of the beam—I; breakage of the layers in the lower part of the beam caused by a rapid deflection increase—II (a result of the “collapse” of the upper part of the beam).

The first evident “mass” fiber fracture takes place only right after the point of load capacity collapse, corresponding to the basic laminate failure. The phenomenological description of the classic laminate failure is based on the presence of rigid layers of impregnated reinforcement interleaved with thin layers of the pure matrix, which stick them together. During bending, in the layers close to the upper part of the beam, a stress concentration occurs, caused by the simultaneous operation of two forces. One of latter is the cutting force coming from the mutual shift of the neighboring reinforcement layers (apparent shortening of the upper part of the beam during bending). The other one is the force extending (tearing) the neighboring layers one from the other, caused by the tendency of the upper layer to buckle. This tendency is the result of this layer’s compression due to its apparent shortening during bending. The stress concentration, at a certain point, causes a cohesion break of the thin layer of the matrix and the occurrence of a delamination fracture in a relatively large area. Local buckling and stress concentrations are characteristic phenomena occurring in laminated structures [63,64]. This is also the cause of buckling of the upper reinforcement layer and a rapid loss of a part of the load capacity by the beam. This, in turn, leads to a “collapse” of the beam: a significant deflection increase, which leads to fiber fracture in its lower (stretched) part. The elements of the above description are presented graphically in Figure 6. 

**Figure 7:**
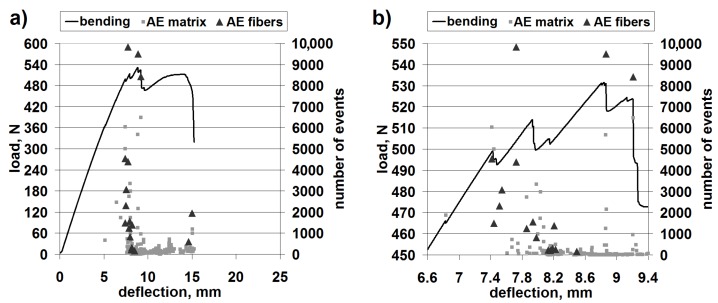
Mechanical curve and separated AE signal for 3D laminate, direction *W*: (**a**) The whole progress; (**b**) Magnification of the crucial fragment.

Figure 7 shows the results for a representative bending test with superimposed AE diagrams, with separated points corresponding to matrix fracture and fiber fracture, for 3D laminate, direction *W*. 

The obtained load capacity of the beam suggests a bending strength of the order of 250–260 MPa, which constitutes a comparable value to that of the classic laminate. 

**Figure 8:**
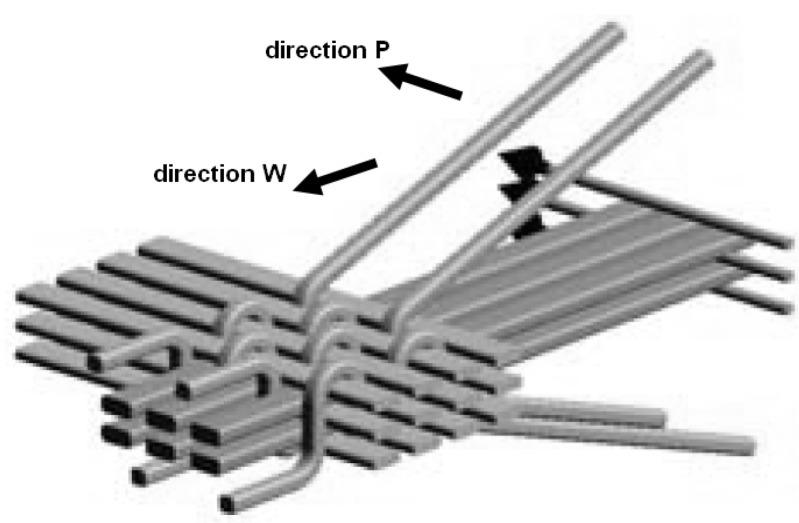
Schematic diagram of the reinforcement structure of a 3D non-crimp fabric (NCF) fabric by 3TEX [65].

Differently from the case of the classic laminate, the 3D-*W* laminate exhibits a larger contribution of the fiber fracture in the basic failure. However, the beginning of the failure process is evidently connected with the resin fracture (Figure 7, at about 6.9 mm of deflection); this is undoubtedly delamination. In this type of laminate, the structure (non-crimp fabric (NCF)) is different from that in the classic laminate. It resembles a lattice made from crosswise fiber strands connected by strands of a translaminar interweave (Figure 8). 

**Figure 9:**
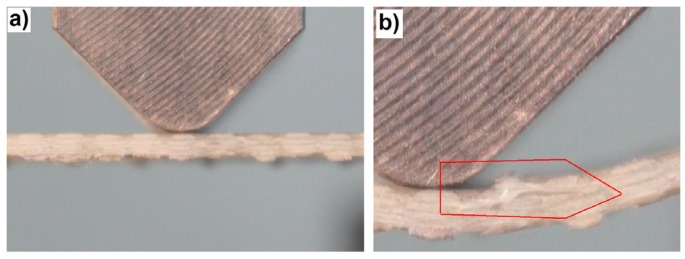
Photograph showing the main failure in the 3D laminate, direction *W*: (**a**) Beginning of the bending test; (**b**) 60 s of the test, right after the occurrence of the main failure.

This first acoustic event corresponds to the slight drop of load capacity on the bending curve. However, practically all of the following load capacity drops on the curve correspond both to the matrix fracture and the fiber fracture. The macroscopic observation of the failure is shown in Figure 9. 

This shows that the failure progress is similar to the one in the classic laminate; however, next to the extensive delamination fracture, there is also a significant damage of the fibers in the upper layers. The significant contribution of the fibers in the basic laminate failure progress during bending is proven by the shape of the mechanical curve, where there is no rapid drop of the load capacity (as in the classic laminate; Figure 4). Up to the moment of a very large deformation (about 15 mm), after each load capacity drop, one observes the load increase, which is characteristic for the fiber contribution to load carrying. This is a very beneficial (“quasi-plastic”) model of the failure progress. 

**Figure 10:**
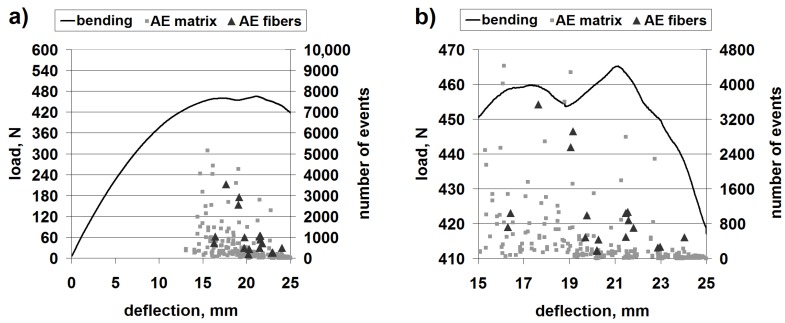
Mechanical curve and separated AE signal for 3D laminate, direction *P*: (**a**) The whole progress; (**b**) Magnification of the crucial fragment.

Figure 10 shows the results of a representative bending test with superimposed AE diagrams, with separated points corresponding to matrix fracture and fiber fracture, for the 3D laminate, direction *P*. 

The obtained load capacity of the beam points to a bending strength of the order of 230 MPa, which constitutes a value comparable to that of the 3D-*W* laminate and the classic laminate. Therefore, the anisotropy of the structure causes only a slight anisotropy of the mechanical properties of the 3D laminate. 

**Figure 11:**
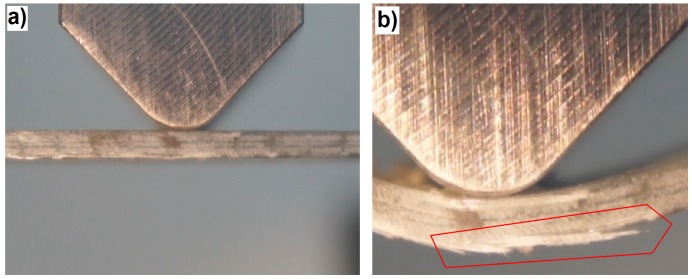
Photograph showing the main failure in the 3D laminate, direction *P*: (**a**) Beginning of the bending test; (**b**) 73 s of the test, right after the occurrence of the main failure.

In the bending diagrams, one can see a beneficial type of failure, similar to (or even slightly milder than) that for the 3D-*W* laminate. However, at the initial stage of the basic failure (from about 13 mm of deflection), the main mechanism is matrix fracture. Fiber fracture takes place only at the moment of a partial load capacity loss of the beam. Such behavior proves a significant contribution of the translaminar interweave strands in the work of the 3D laminate, during bending in direction *W*. The lack of this action in direction *P* results in a milder model of failure, yet, at the same time, laminate weakening (lower maximal strain); a similar regularity was observed for stitched laminates [55]. The lattice structure and the presence of relatively large cracks between the fiber bands (Figure 8) result in a locally-facilitated bending of the laminate in direction *P* (local reduction of the section height). Figure 11 shows practically no delamination in the upper part of the beam, whereas the main failure is the extensive fracture in the lower part. 

Therefore, the increased possibilities of the deformation of the 3D laminate in direction *P* “replace” the collapse resulting from delamination in the classic and 3D-*W* laminates. 

The above results suggest that the method of signal separation performed by means of the event energy is effective in the analyzed case. However, as was mentioned earlier, it is impossible to be used to analyze the fracture of small amounts of fibers, which makes the assessment of the initial stage of the test difficult, before the occurrence of the main area of failure. Therefore, this is not a universal method, and one should initially assess whether the conditions of the given test and the particular analyzed material are suitable for it. It is, however, the only analytical tool in the case when it is impossible to separate the signal with the use of frequency. 

In order to verify the above results of the AE analysis, an additional analysis of the vibration signal registered in the bending tests was performed. 

### 3.2. Vibrations Signal Results 

**Figure 12:**
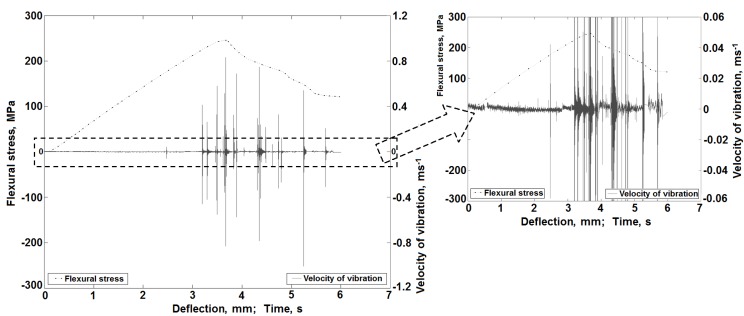
Characteristics of the vibration rate changes during a bending test for the classic laminate sample [53].

**Figure 13:**
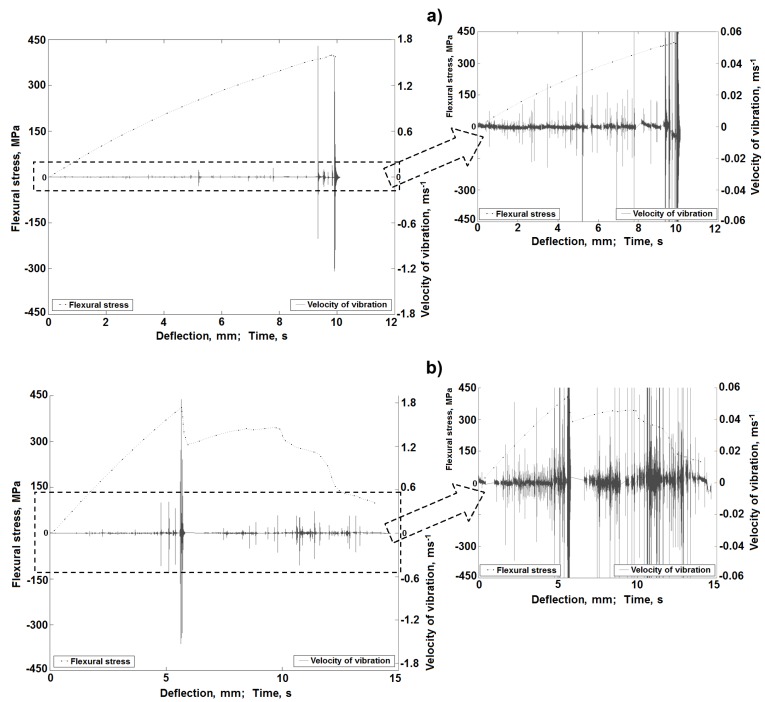
Characteristics of the vibration rate changes during a bending test for a 3D laminate sample: (**a**) Load direction along translaminar interweave strands (*W*); (**b**) Load direction perpendicular to translaminar interweave strands (*P*) [53].

During a separate series of static bending tests, the vibration rate signals were registered (Figure 1b). Mechanical curves with superimposed diagrams of the vibration rate of the sample material were obtained. Exemplary, representative results of these measurements for the classic laminate are included in Figure 12, whereas those for the 3D laminates in Figure 13. Part of this result was formerly presented in [53]. 

As can be observed in the diagrams, together with the increase of the sample deflection, local increases of the vibration signal amplitude occur. In the first deflection range, up to about 2.4 mm for the classic laminate, up to about 5 mm for the 3D-*W* laminate and about 4.5 mm for the 3D-*P* laminate, we can observe slight energy changes. These changes evidently prove the occurrence of laminate damage effects. The latter can be local matrix fractures or fractures of single fibers in the external, most pressed or stretched areas. Such effects were not visible in the AE signal, which proves the high precision of the vibration registration method. Only beyond these deflection values can we observe significant local vibration amplitude increases with local extremes, which overlap with the ranges of the local extremes on the bending curves, thus overlapping with the local effects of sample damage. 

**Figure 14:**
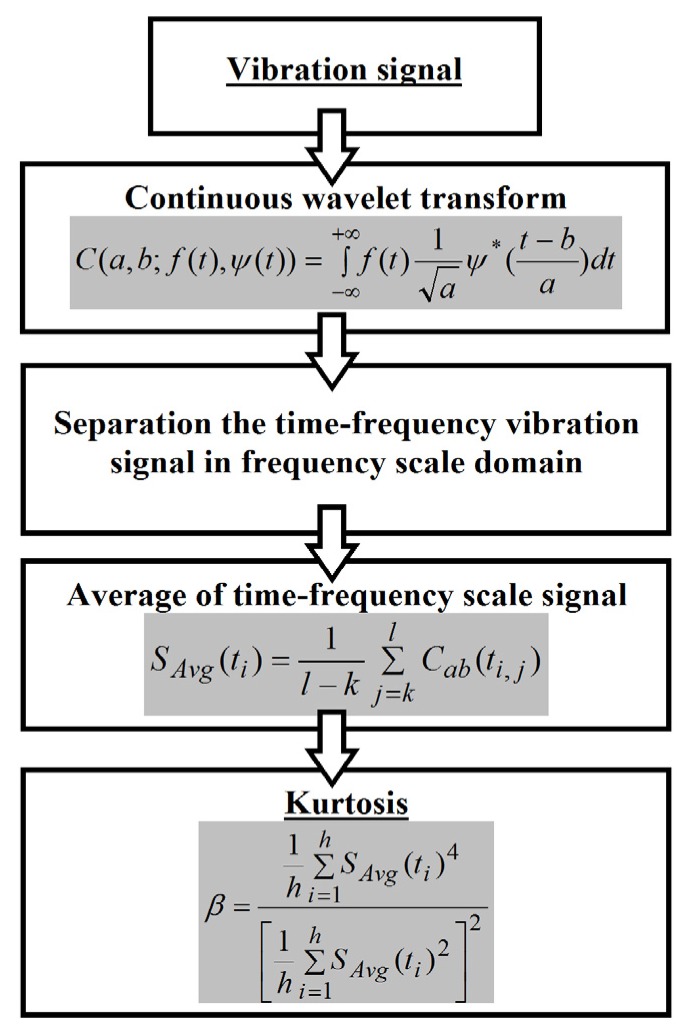
Algorithm of the performed calculations for the determination of kurtosis (details in [66]).

In their earlier works [53,54], the authors proved the usefulness of the registered vibration signals, among others, with the application of filtration and spot measures, where a frequency division of the vibration signal into the matrix fracture data and the fiber fracture data was obtained. On the basis of the further tests performed by the authors, [66] presents a new method of vibration signal separation in classic laminate samples. It considers signal processing with the use of a continuous wavelet transform [67], signal separation in the domain of the frequency scale [68] and, next, calculation of the averaged signals and kurtosis, as an energy spot measure of the information included in the vibration signal. The schematic algorithm used in this method is presented in Figure 14. 

**Figure 15:**
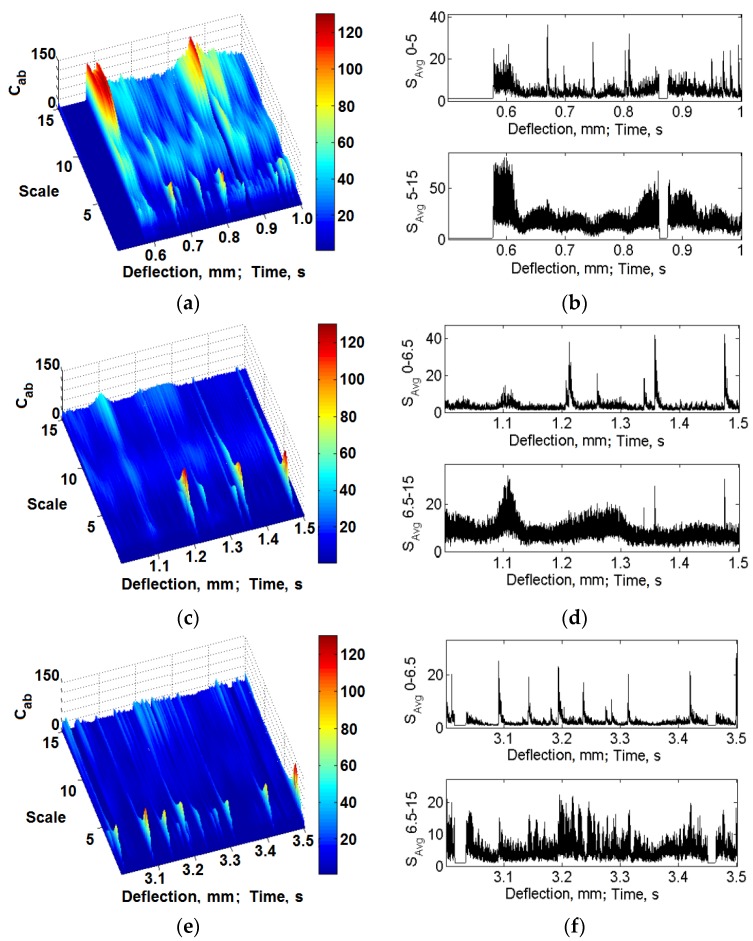
Exemplary time frequency scale distributions and averaged values, initial deflection: (**a**–**b**) Classic laminate; (**c**–**d**) 3D-*W* laminate; (**e**–**f**) 3D-*P* laminate.

**Figure 16:**
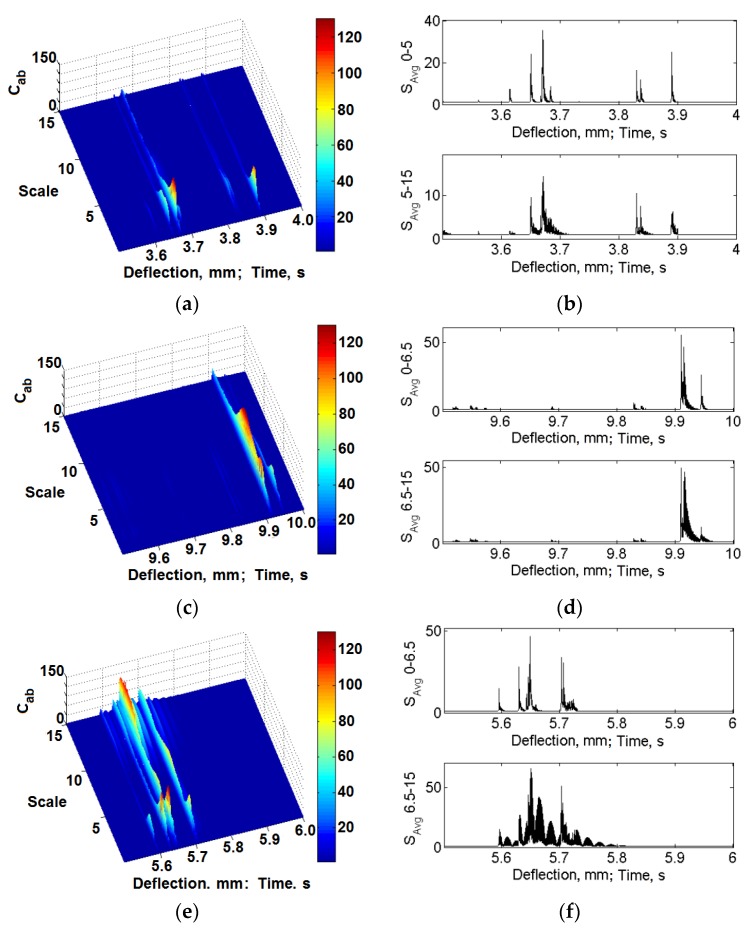
Exemplary time frequency scale distributions and averaged values, deflection range at which significant laminate degradation damage occurs: (**a**–**b**) Classic laminate; (**c**–**d**) 3D-*W* laminate; (**e**–**f**) 3D-*P* laminate.

Figure 15 and Figure 16 include exemplary results of the calculations with the use of the presented method for the classic laminate and the 3D laminate (bent in the *W* and *P* directions). The selected results present the time frequency scale distributions and the calculated mean values in the selected scale ranges for the initial deflection stage, as well as for the deflection at which the basic laminate damage occurs. 

It was found that slight energy changes, within the scale range of 0–5 for the classic laminate and 0–6.5 for the 3D laminates, in the distributions time-vibration signal frequency scale are visible, which are connected to the first symptoms of laminate matrix damage (Figure 15). Together with the increase of sample deflection, the change in the vibration energy proceeds. In the range of the sample’s maximal values of bending stress and at the moment of its fracture, one can observe a clear significant increase of the vibration signal energy. In the case of the sample deflection accompanied by a clear laminate failure, we observe an energy increase within the scale range of >5 in the distributions, for the classic laminate, and >6.5, for the 3D laminates (Figure 16). These changes can be attributed to the extensive damage of the matrix and the significant damage of the laminate fibers. 

Taking into account the above, averaging of the time frequency scale distributions was performed in the following ranges: 0–5 and 5–15 for the classic laminate and 0–6.5 and 6.5–15 for the 3D laminates. The results of these calculations are included in Figure 15b,d,f and Figure 16b,d,f. They suggest local increases of the vibration energy according to the regularities described above, observed in the time frequency scale distributions. Considering this, one can state that, in the scale range of 0–5 for the classic laminate and 0–6.5 for the 3D laminates, we observe laminate matrix damage, whereas fiber damage is observed in the scale range of 5–15 for the classic laminate and 6.5–15 for the 3D laminates. 

**Figure 17:**
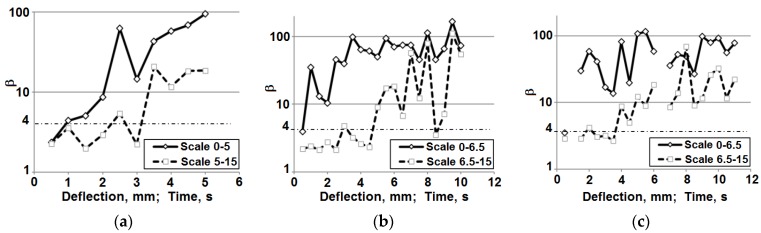
Kurtosis: (**a**) Classic laminate; (**b**) 3D laminate, direction *W*; (**c**) 3D laminate, direction *P*.

For the purpose of a quantitative description of the qualitative changes taking place in the processed vibration signals, in the further calculations, the kurtosis was determined [53,69]. Its calculation was based on the averaged signals in the scale range of 0–5 and 5–15 for the classic laminate and 0–6.5 and 6.5–15 for the 3D laminates (Figure 15b,d,f and Figure 16b,d,f). The results of these calculations are included in Figure 17. 

As can be inferred from the calculations, the value of the kurtosis above four proves the occurrence of damage in the analyzed composites. In the scale range of 0–5 for the classic laminate and 0–6.5 for the 3D laminates, we observe an increase of the measure value already at the initial stage of the bending test, which proves the described above assumptions of the initial, low-energy, damage, most probably of the laminate matrix. The kurtosis calculations in the range of 5–15 for the classic laminate and 6.5–15 for the 3D laminates suggest a measured increase only at a significant sample deflection, which proves the occurrence of a high-energy fiber fracture in the samples and an accompanying lower energy matrix fracture. 

## 4. Conclusions

1. The applied innovative method of separating the acoustic emission signal coming from fiber fracture and the one coming from matrix fracture with the use of the acoustic event energy proves very good in the analysis of the main laminate failure. A problematic issue is the detection of a simultaneous fracture of small amounts of elementary fibers, which makes the separative analysis impossible at an earlier stage of failure. At the same time, emission remains a good tool for the detection of initial failure processes in the case when there is no need to separate the signal.
2. The method of vibration signal analysis is more precise than the method of acoustic emission, due to its higher frequency of sampling and higher sensitivity of the measuring system. It is very useful in the detection and analysis of the signal originating from the effects occurring in the initial stage of material failure, which significantly precedes the main failure. In this respect, vibration analysis is a valuable complementation, or alternative, for acoustic emission.
3. The energy approach to signal separation can be effectively implemented with the use of the kurtosis of the signal progress.
4. In the comparison of the test results obtained in separate bending tests by the two different methods of signal registration and analysis, one can state that the results are similar, both with respect of the general laminate failure progress and the occurrence of separated signals of fiber fracture and matrix fracture.
5. The strength of the 3D laminate is comparable with that of the classic laminate. This results from the overlap of the beneficial “non-crimp” effect and the negative effect of the “lattice” construction.
6. The strength of the 3D laminate is slightly lower in the direction perpendicular to the translaminar interweave strands than in the direction parallel to them.
7. The 3D laminate is characterized as having a much higher deformability than the classic laminate, especially in the direction perpendicular to the translaminar interweave strands.
8. The failure progress of the 3D laminate is “safer” than that of the classic laminate, especially in the direction perpendicular to the translaminar interweave strands. It exhibits a milder failure model, with less rapid load capacity drops. The results of the acoustic emission also show a higher fraction of fiber fracture, already before the occurrence of the basic material failure. This is a very beneficial failure model, which proves the occurrence of effort, that is the work of the fibers during the load shift.
